# Timing of initiation of intra‐aortic balloon pump in patients with acute myocardial infarction complicated by cardiogenic shock: A meta‐analysis

**DOI:** 10.1002/clc.23264

**Published:** 2019-09-11

**Authors:** Kongyong Cui, Shuzheng Lyu, Hong Liu, Xiantao Song, Fei Yuan, Feng Xu, Min Zhang, Mingduo Zhang, Wei Wang, Dongfeng Zhang, Jinfan Tian, Yunfeng Yan, Kuo Zhou, Lingxiao Chen

**Affiliations:** ^1^ Department of Cardiology Beijing Anzhen Hospital, Capital Medical University and Beijing Institute of Heart, Lung and Blood Vessel Diseases Beijing China

**Keywords:** acute myocardial infarction, cardiogenic shock, intra‐aortic balloon pump, survival, timing

## Abstract

**Background:**

For patients with acute myocardial infarction (AMI) complicated by cardiogenic shock (CS) undergoing primary percutaneous coronary intervention (PCI), the optimal timing of the initiation of intra‐aortic balloon pump (IABP) therapy remains unclear. Therefore, we performed the first meta‐analysis to compare the outcomes of IABP insertion before vs after primary PCI in this population.

**Methods:**

Electronic databases of PubMed, EMBASE, and Cochrane Library were comprehensively searched from inception to April 1, 2019, to identify the eligible studies. The main outcomes were short‐term (in‐hospital or 30 days) and long‐term (≥ 6 months) mortality. In addition, pooled analysis of risk‐adjusted data were also performed to control for confounding factors.

**Results:**

Seven observational studies and two sub‐analysis of randomized controlled trials involving 1348 patients were included. Compared to patients inserted IABP after PCI, patients who received IABP therapy before primary PCI had similar risks of short‐term (odds ratio [OR] 0.88, 95% CI 0.49 to 1.59) and long‐term (OR 0.99, 95% CI 0.58 to 1.68) all‐cause mortality. Moreover, a pooled analysis of risk‐adjusted data also found similar effects of the two therapies on short‐term (OR 0.65, 95% CI 0.34 to 1.25) and long‐term (OR 0.68, 95% CI 0.17 to 2.72) mortality. Besides, no significant difference was found between the two groups with respect to reinfarction, repeat revascularization, stroke, renal failure, and major bleeding.

**Conclusions:**

The timing of the initiation of IABP therapy does not appear to impact short‐term and long‐term survival in patients with AMI complicated by CS undergoing primary PCI.

## 1 INTRODUCTION

In patients with acute myocardial infarction (AMI), 6%‐9% can be affected by cardiogenic shock (CS) and the mortality rate is close to 50% during hospitalization.^1^ Despite adoption of early revascularization strategies, CS remains the leading cause of death in this population.^2^ Furthermore, supportive drug treatments with inotropes and vasopressors bring no benefit to patients. Cardiologists hope that mechanical circulatory support will improve clinical outcomes in this population.^3^, ^4^ The intra‐aortic balloon pump (IABP) becomes the first and most widely used device due to its ability to reduce afterload and improve coronary blood flow.^4^, ^5^, ^6^, ^7^ However, recent meta‐analyses and the landmark randomized controlled trial did not show a beneficial effect of IABP in patients with AMI complicated by CS.^8^, ^9^, ^10^, ^11^ Thus, the recommendations of IABP therapy have been reduced both in the American and European guidelines.^12^, ^13^ Nonetheless, the lack of efficacy of IABP usage might be partly influenced by the timing of initiation of IABP therapy, that is, before or after primary percutaneous coronary intervention (PCI). Nevertheless, almost all studies comparing the sequence of IABP and primary PCI are of a small scale, and current trials have shown conflicting results. Thus, we conducted this meta‐analysis to identify the optimal timing of the initiation of IABP in patients with AMI complicated by CS undergoing primary PCI.

## 2 METHODS

This study was performed based on the preferred reporting items for systematic reviews and meta‐analyses (PRISMA) and meta‐analysis of observational studies in epidemiology (MOOSE) statements.^14^, ^15^

### 2.1 Search strategy

Two independent investigators (Lingxiao Chen and Kuo Zhou) searched the electronic databases of PubMed, EMBASE, and Cochrane Library from inception to April 1, 2019, to identify the pertinent English articles regarding the IABP inserted before vs after primary PCI for the treatment of AMI complicated by CS. The following medical subject headings and search terms were used: “acute myocardial infarction,” “cardiogenic shock,” “before primary percutaneous coronary intervention,” “after primary percutaneous coronary intervention,” and “timing.” In addition, the references of the identified articles and relevant reviews were examined to include other potentially eligible studies.

### 2.2 Study selection

Studies satisfying the following criteria were eligible: (a) patients who were diagnosed with CS from AMI; (b) studies that compared the strategy of IABP insertion before vs after primary PCI; and (c) studies that assessed the endpoints of interest. The selection was conducted by scanning titles and/or abstracts, and full‐text reviews were performed for further analysis. When several reports overlapped, we selected the largest and the latest one. The studies were reviewed by two independent investigators (Jinfan Tian and Yunfeng Yan) to determine whether they met the inclusion criteria. Any disagreements were resolved through discussion with a third investigator (Dongfeng Zhang).

### 2.3 Data extraction and quality assessment

For each eligible study, three authors (Fei Yuan, Mingduo Zhang, and Wei Wang) independently extracted the following data through a standardized form: first author, year of publication, study design, quality indicators, baseline as well as procedural characteristics, and clinical outcomes. Discrepancies were resolved by consensus. The primary endpoint was short‐term mortality (in‐hospital or 30 days). Long‐term mortality (≥ 6 months), reinfarction, stroke, repeat revascularization, acute renal failure, and major bleeding were the secondary outcomes. Deaths were classified as either cardiac or noncardiac, and classifications of other outcomes were in agreement with the included studies.

The methodological quality of the observational studies was assessed using the Newcastle Ottawa Scale.^16^ Studies with a Newcastle‐Ottawa score ≥ 6 (maximum, 9) were considered high quality. In addition, the quality of randomized controlled trials (RCTs) were assessed using the Cochrane risk of bias tool.^17^

### 2.4 Statistical analysis

The present study used Review Manager 5.3 (The Cochrane Collaboration, The Nordic Cochrane Centre, Copenhagen, Denmark) and Stata/SE12.0 (StataCorp, College Station, Texas) for data analysis. All results were presented as odds ratios (ORs) and 95% confidence intervals (CIs). Potential heterogeneity was evaluated with the I^2^ statistic, and a value >50% was defined as statistical heterogeneity. For all comparisons, the DerSimonian and Lair random‐effects model was used to account for the wide range of methodological variability across the studies.

Pooled analysis of risk‐adjusted data were performed to control for confounding factors, and to test the sensitivity of the short‐term and long‐term mortality. The adjusted variables are listed in Table S1. In addition, sensitivity analysis was conducted by reanalyzing the results of studies that enrolled patients presented with ST‐segment elevation myocardial infarction (STEMI) or published in full text. In case of significant heterogeneity, sensitivity analysis was also conducted by omitting one study in each turn to test the influence of single trial. Meta‐regression analysis was carried out to assess patient characteristics with the primary endpoint, that is, male, current smoker, diabetes mellitus, hypertension, and culprit vessel of left anterior descending coronary artery. The risk of potential publication bias was assessed by the Begg's and Egger's tests.^18^, ^19^ When there was an indication of publication bias from the statistical tests, we used the trim and fill method to evaluate the influence of potentially unpublished studies on the summary estimates. All statistical tests were two‐sided and were considered to be statistically significant at *P* < .05.

## 3 RESULTS

### 3.1 Eligible studies

The comprehensive search yielded 1093 potentially relevant articles; after exclusion of duplicates and assessment of titles and/or abstracts, 29 articles were chosen for complete review. Finally, nine studies including 1348 patients met our inclusion criteria, published between 2005 and 2017^20^, ^21^, ^22^, ^23^, ^24^, ^25^, ^26^, ^27^, ^28^ (Figure S1).

The main characteristics of the included studies are presented in Table 1 and quality assessment results are described in Tables S2 and S3. Seven studies were observational studies,^21^, ^22^, ^23^, ^24^, ^25^, ^26^, ^27^ and the remaining two were sub‐analysis of randomized controlled trials.^20^, ^28^ Five of the eligible studies only enrolled patients who presented with STEMI,^22^, ^23^, ^24^, ^25^, ^27^ and the remaining four included patients with non‐STEMI.^20^, ^21^, ^26^, ^28^ Two studies were abstract slides from conference proceedings. Overall, eight studies reported short‐term mortality (in‐hospital and 30‐days),^20^, ^21^, ^22^, ^23^, ^24^, ^25^, ^26^, ^28^ while four studies reported long‐term mortality (≥ 6 months).^23^, ^25^, ^27^, ^28^ In addition, five^21^, ^22^, ^23^, ^24^, ^26^ and three^23^, ^27^, ^28^ studies reported multivariable‐adjusted data of short‐term and long‐term mortality, respectively.

**Table 1:** The methodology and the population characteristics of studies

Study	No. patients^a^	Period	Region	Design, center	Inclusion criteria	Exclusion criteria	Adjusted method	Follow‐up duration
Thiele 2005	9/11	2000‐2003	Germany	Sub‐analysis of RCT, single	AMI with CS	Age > 75 y, mechanical complication, shock >12 h, right heart failure, sepsis, significant aortic regurgitation, severe cerebral damage, resuscitation >30 min, severe peripheral vascular disease	NA	30 d
Abdel‐Wahab 2010	26/22	2005‐2008	Germany	Retrospective registry, single	AMI with CS due to left ventricular failure	Mechanical complication, isolated right ventricular infarction, shock due to other causes, > 24 h after primary PCI	Multivariable adjusted	In‐hospital
Sjauw 2012	59/140	1997‐2005	Netherlands	Prospective registry, single	STEMI with CS	NA	Propensity‐score adjusted	30 d
Cheng 2013	87/86	2000‐2009	Netherlands	Retrospective registry, single	STEMI with CS	CS developed during primary PCI or hospitalization	Multivariable adjusted	5 y
Bergh 2014	72/67	2004‐2008	Sweden	Prospective registry, single	STEMI with CS	NA	Propensity‐score adjusted	30 d
Negi 2014	76/98	NA	United States	Retrospective study, single	STEMI with CS	NA	NA	1 y
Schwarz 2016	49/53	2005‐2010	Germany	Retrospective registry, single	AMI with CS due to left ventricular failure	No spontaneous circulation, mechanical complication, isolated right ventricular infarction, shock due to other causes, > 24 h after primary PCI	Multivariable adjusted	In‐hospital
Yuan 2016	106/112	2008‐2014	China	Prospective study, single	STEMI with CS	Incomplete data	Multivariable adjusted	1 y
Fuernau 2017	33/242	2009‐2012	Germany	Sub‐analysis of RCT, multi	AMI with CS	Resuscitation >30 min, no spontaneous circulation, coma, mechanical complication, shock >12 h, massive pulmonary embolism, severe peripheral arterial disease or aortic regurgitation, age > 90 years, shock due to other causes	Multivariable adjusted	1 y

Abbreviations: AMI, acute myocardial infarction; CS, cardiogenic shock; IABP, intra‐aortic balloon pump; NA, not applicable; PCI, percutaneous coronary intervention; RCT, randomized controlled trial; STEMI, ST‐segment elevation myocardial infarction.

a Data are expressed as IABP before primary PCI/ IABP after primary PCI.

As presented in Table 2, baseline characteristics of the patients were similar between the two treatment strategies, except that dyslipidemia was more common in patients who received IABP insertion before primary PCI than the control group (48.3% vs 38.7%).

**Table 2:** Baseline characteristics of the patients

Study	Age, years	Male (%)	Current smoker (%)	Diabetes mellitus (%)	Hypertension (%)	Dyslipidemia (%)	Previous MI (%)	LVEF (%)	Systolic blood pressure, mmHg	Multivessel disease (%)	Culprit vessel of LAD (%)	IABP support time, hours
Thiele 2005	NA	NA	NA	NA	NA	NA	NA	NA	NA	NA	NA	NA
Abdel‐Wahab 2010	70/71	88.5/72.7	42.3/40.9	50.0/45.5	69.2/63.6	57.7/54.5	34.6/40.9	23.5/23.2	109/105	NA	46.2/45.5	43/47
Sjauw 2012	65.1/64.6	79.7/63.6	30.5/38.6	15.3/19.3	37.3/33.6	25.4/19.3	28.8/30.7	NA	109.7/104.8	64.4/62.1	55.9/61.4	NA
Cheng 2013	65/64	79.3/77.9	24.1/29.1	17.2/16.3	29.9/27.9	56.3/47.7	29.9/18.6	NA	75/79	NA	66.7/55.8	NA
Bergh 2014	66/66	69.4/76.1	30.6/26.9	29.2/14.9	36.1/32.8	NA	13.9/13.4	33/35	80/79	77.8/74.6	NA	20/24
Negi 2014	59/61	63.2/74.5	NA	NA	NA	NA	NA	NA	NA	NA	88.2/88.8	56/51
Schwarz 2016	70.6/69.7	73.5/66.0	32.7/32.1	51.0/41.5	73.5/71.7	61.2/52.8	34.7/22.6	24.2/25.2	NA	NA	NA	37/45
Yuan 2016	63.1/65.2	64.2/63.4	44.3/47.3	30.2/29.1	36.8/38.4	46.2/46.4	10.4/14.3	NA	76.5/75.7	24.5/29.5	48.1/49.1	NA
Fuernau 2017	NA	NA	NA	NA	NA	NA	NA	NA	NA	NA	NA	NA
Summary	64.6/64.9	71.8/69.6	33.8/36.7	28.8/24.4	41.9/39.2	**48.3/38.7**	22.6/21.9	28.4/29.5	84.9/87.9	50.6/48.7	63.0/62.4	38.7/41.8

*Note*: Data are expressed as IABP inserted before primary percutaneous coronary intervention/IABP inserted after primary percutaneous coronary intervention. Bold values that dyslipidemia was more common in patients who received IABP insertion before primary PCI than the control group (48.3% vs 38.7%), while other baseline characteristics of the patients were similar between the two groups.

Abbreviations: IABP, intra‐aortic balloon pump; LAD, left anterior descending coronary artery; LVEF, left ventricular ejection fraction; MI, myocardial infarction; NA, not applicable.

### 3.2 Primary endpoint

**Figure 1:**
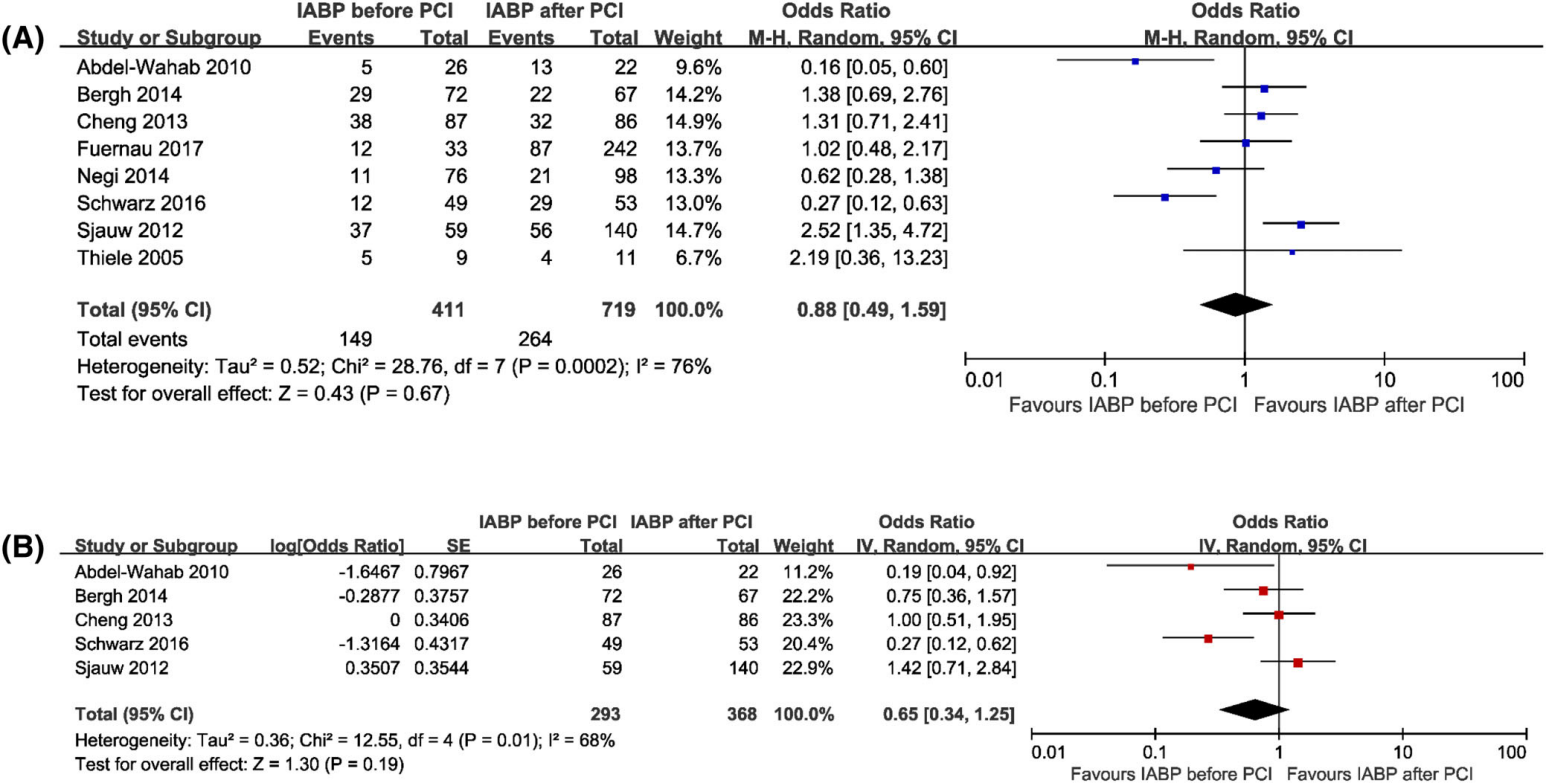
Forest plots comparing short‐term mortality for patients with acute myocardial infarction complicated by cardiogenic shock undergoing IABP insertion before or after primary percutaneous coronary intervention. A, Unadjusted short‐term mortality. B, Adjusted short‐term mortality. CI, confidence interval; IABP, intra‐aortic balloon pump; PCI, percutaneous coronary intervention

In summary, short‐term death occurred in 149 patients (36.3%) in the IABP inserted before primary PCI group compared with 264 patients (36.7%) in the IABP inserted after primary PCI group. As shown in Figure 1A, short‐term mortality was comparable between the two treatment strategies (OR 0.88, 95% CI 0.49 to 1.59, *P* = .67), with significant heterogeneity across studies (I^2^ = 76%, *P* = .0002). Sensitivity analysis indicated that no significant difference was found between the two groups when studies that enrolled patients with STEMI (OR 1.34, 95% CI 0.79 to 2.29, I^2^ = 60%) (Figure S2) or published in full text (OR 0.90, 95% CI 0.40 to 2.00, I^2^ = 81%) (Figure S3) were analyzed. In addition, sensitivity analysis conducted by the removal of any single trial showed that it did not essentially affect the overall pooled estimate of short‐term mortality, whereas the heterogeneity existed consistently across the studies (I^2^ > 50%). Moreover, a pooled analysis of risk‐adjusted data also demonstrated similar effects of the two therapies on short‐term mortality (OR 0.65, 95% CI 0.34 to 1.25, *P* = .19, I^2^ = 68%) (Figure 1B). After removing the study by Schwarz et al., the statistical heterogeneity of adjusted short‐term mortality no longer existed (OR 0.86, 95% CI 0.49 to 1.51, I^2^ = 47%).

### 3.3 Secondary endpoints

**Figure 2:**
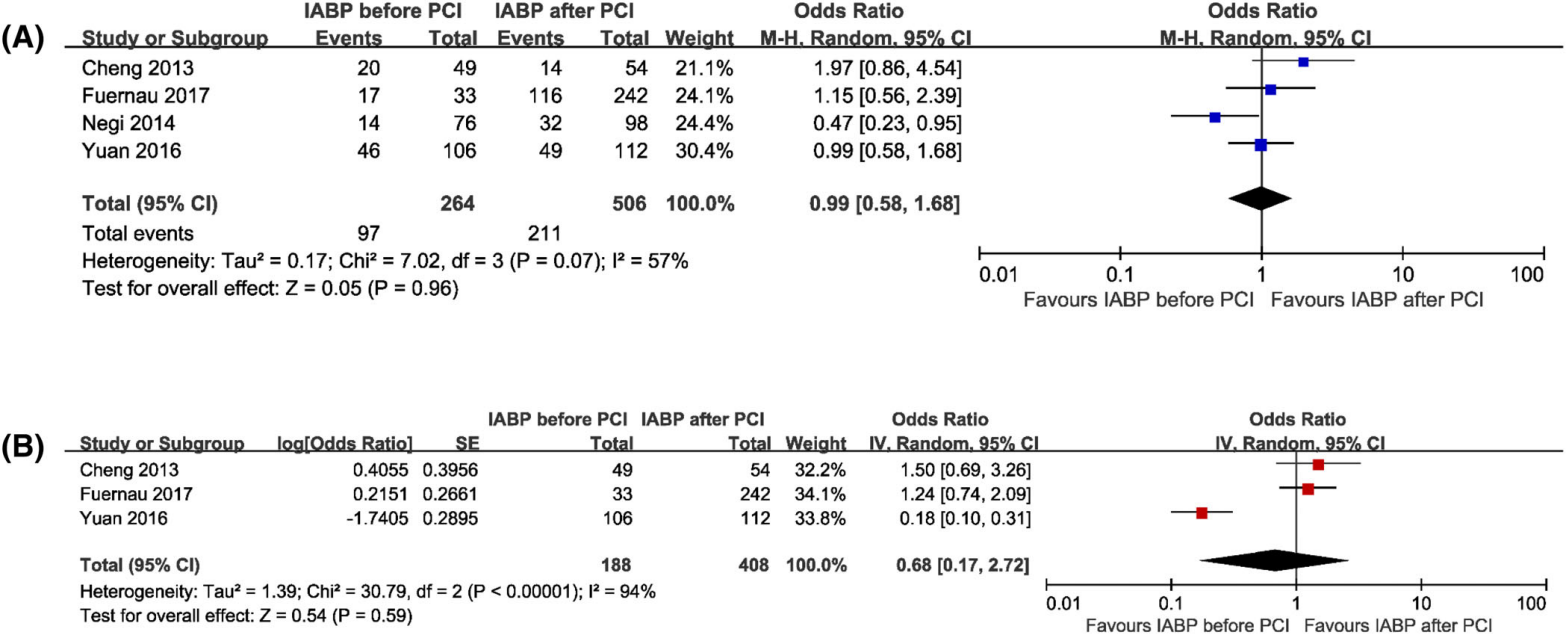
Forest plots comparing long‐term mortality for patients with acute myocardial infarction complicated by cardiogenic shock undergoing IABP insertion before or after primary percutaneous coronary intervention. A, Unadjusted short‐term mortality. B, Adjusted short‐term mortality. CI, confidence interval; IABP, intra‐aortic balloon pump; PCI, percutaneous coronary intervention

In the pooled estimate, the initiation of IABP therapy before primary PCI had similar risk of long‐term mortality compared to that of inserted after primary PCI based on both unadjusted data (OR 0.99, 95% CI 0.58 to 1.68, *P* = .96, I^2^ = 57%) (Figure 2A) and risk‐adjusted data (OR 0.68, 95% CI 0.17 to 2.72, *P* = .59, I^2^ = 94%) (Figure 2B). After removing the study by Negi et al., the heterogeneity of long‐term mortality no longer existed (OR 1.19, 95% CI 0.81 to 1.75, I^2^ = 0%). Besides, the heterogeneity of adjusted long‐term mortality disappeared after excluding the study by Yuan et al. (OR 1.32, 95% CI 0.85 to 2.03, I^2^ = 0%).

**Figure 3:**
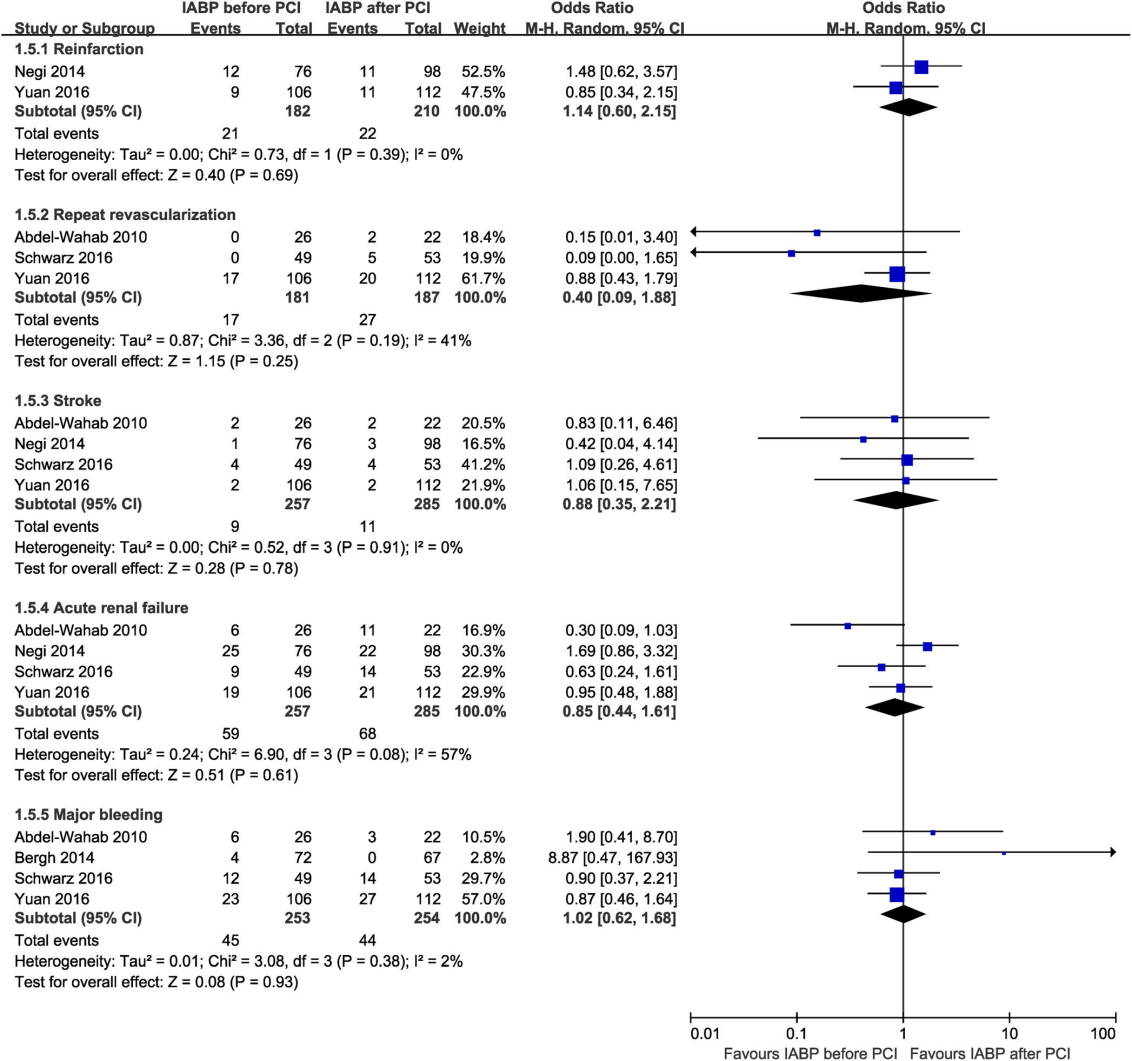
Forest plots comparing reinfarction, repeat revascularizaton, stroke, acute renal failure, and major bleeding for patients with acute myocardial infarction complicated by cardiogenic shock undergoing IABP insertion before or after primary percutaneous coronary intervention. CI, confidence interval; IABP, intra‐aortic balloon pump; PCI, percutaneous coronary intervention

No significant difference was found between the two groups in terms of reinfarction (OR 1.14, 95% CI 0.60 to 2.15, *P* = .69, I^2^ = 0%), repeat revascularization (OR 0.40, 95% CI 0.09 to 1.88, *P* = .25, I^2^ = 41%), stroke (OR 0.88, 95% CI 0.35 to 2.21, *P* = .78, I^2^ = 0%), acute renal failure (OR 0.85, 95% CI 0.44 to 1.61, *P* = .61, I^2^ = 57%), and major bleeding (OR 1.02, 95% CI 0.62 to 1.68, *P* = .93, I^2^ = 2%) (Figure 3). The heterogeneity of acute renal failure no longer existed when the study by Abdel‐Wahab et al., (OR 1.07, 95% CI 0.62 to 1.84, I^2^ = 36%) or by Negi et al., (OR 0.66, 95% CI 0.36 to 1.20, I^2^ = 23%) was removed.

### 3.4 Meta‐regression analysis and publication bias

Meta‐regression analysis showed significant association between patient characteristics of diabetes mellitus (regression coefficient − 0.07, 95% CI −0.21 to −0.02, *P* = .02) or hypertension (regression coefficient − 0.05, 95% CI −0.09 to −0.01, *P* = .04) and the short‐term mortality. No interaction was found between male (*P* = .51), current smoker (*P* = .42), or culprit vessel of left anterior descending coronary artery (*P* = .79) and the primary endpoint of short‐term mortality.

In addition, the assessment of the funnel plot was performed, and no publication bias was found for the outcomes except for major bleeding (Egger's test, *P* = .03; Begg's test, *P* = .09). One study was added with the trim and fill method, and the risk of major bleeding remained similar between the two treatment strategies (OR 1.00, 95% CI 0.53 to 1.88) (Figure S4).

## 4 DISCUSSION

This is the first meta‐analysis comparing the two treatment strategies of IABP inserted before and after primary PCI in patients with AMI complicated by CS. Our data suggest that the timing of initiation of IABP therapy does not have an effect on short‐term and long‐term survival in this population. Besides, the risks of reinfarction, repeat revascularization, stroke, acute renal failure, and major bleeding were similar between the two groups.

Since 1968, the IABP has been used for mechanical cardiac assistance in patients with CS.^29^ In theory, the deflation during systole reduces ventricular afterload and helps the ventricle push blood into the aorta, while the inflation during diastole enhances coronary artery perfusion and promotes blood flow to systemic organs.^5^ Based on pathophysiological considerations and benefits observed in nonrandomized studies in the pre‐PCI era, previous American Heart Association/American College of Cardiology and European Society of Cardiology guidelines gave the use of IABP a class I recommendation for the management of AMI patients with CS. Nevertheless, the results of recent meta‐analyses and the landmark intraaortic balloon pump in cardiogenic shock II (IABP‐SHOCK II) trial have cast doubt on the efficacy of IABP because IABP support does not reduce short‐term and long‐term mortality in patients with AMI complicated by CS.^8^, ^9^, ^10^, ^11^ Although the beneficial effect of IABP therapy on hemodynamic parameters has not translated to a beneficial effect on mortality in these studies, this result may be affected by multiple other factors. For example, 10%‐30% patients with CS in the non‐IABP group received emergency IABP insertion, and the frequent crossover in the randomized controlled trials definitely had an impact on the results according to the intention‐to‐treat principle.^11^, ^30^ In addition, only 13.4% patients in the IABP group inserted the balloon pump before revascularization in the IABP‐SHOCK II trial, and the timing of initiation of IABP therapy might be also of great importance in this setting.^11^

Over the last decade, the debate about the timing of IABP insertion has never stopped, and clinical trials have produced conflicting results. Previous experimental study with animal models of ischemia‐reperfusion demonstrated that unloading the left ventricle with IABP prior to revascularization might provide an additional infarct size reduction. 31, 32 Thereafter, a small population study with 48 patients reported that patients who underwent primary PCI assisted by IABP had a more favorable in‐hospital survival rate than those who received IABP therapy after primary PCI.^21^ Contrariwise, Cheng et al. (n = 173) found that IABP insertion before PCI was associated with a larger infarct size, and no difference was found between the two strategies regarding short‐term and long‐term mortality.^23^ Considering the small sample size of the studies and the controversial results, pooled analysis of the individual data may be informative.

The principal finding of this study is that the timing of IABP insertion that is, before or after primary PCI does not have an effect on the short‐term and long‐term mortality in patients with AMI complicated by CS. It is believed that the early initiation of IABP therapy improves myocardial perfusion and results in significant myocardial salvage than reperfusion alone.^33^ More importantly, hemodynamic stabilization in the setting of cardiogenic shock can prevent the relevant multi‐organ dysfunction or failure.^34^ One possible explanation is that the advantages of early initiation of IABP support are offset by the delay in revascularization associated with the time needed for IABP insertion.^23^ In patients with AMI treated with primary PCI, time to reperfusion determines the extent of reversible and irreversible myocardial injury. However, most of the included studies did not report the data of time delay or door‐to‐balloon time. The study conducted by Yuan et al. indicated an additional delay of 14 minutes in STEMI patients who received IABP therapy before primary PCI compared to those who underwent primary PCI alone (*P* = .04). 27 The study by Abdel‐Wahab et al. found a 35‐minute delay in patients who inserted IABP before primary PCI even without statistical difference.^21^ Although the delay is short, it can still cause greater damage to the microcirculation and myocardium. Therefore, this increased the incidence of major adverse cardiac events, especially in the early stage of AMI.^35^, ^36^ Notably, most of the studies eligible for this meta‐analysis are retrospective observational studies rather than RCTs, and baseline characteristics like dyslipidemia are not comparable between the two groups. Thus, any comparison in outcomes among patients with AMI complicated by CS undergoing IABP insertion before vs after primary PCI is subject to significant confounding. Moreover, the unmeasured confounders between the two mechanical circulatory support strategies might have a significant impact on the results, due to lack of detailed reporting of patient characteristics.

### 4.1 Limitations

Our meta‐analysis presents several limitations that merit attention. First, observational studies were mainly included in our meta‐analysis due to lack of randomized data. This introduced intense heterogeneity and potential bias. Hence, the findings of the present study should be interpreted as hypothesis‐generating only, and could not be overstated. The random‐effect model was used to account for the heterogeneity. Although sensitivity analysis with multivariable‐adjusted data was performed, the potential bias cannot be completely eliminated. Furthermore, meta‐regression analysis found that short‐term mortality might be interfered by baseline characteristics of diabetes mellitus and hypertension. Second, patients with STEMI and non‐STEMI were both enrolled in our meta‐analysis. In this case, sensitivity analysis was conducted by analyzing the results of studies that enrolled patients with STEMI exclusively, and the results were in line with the overall population. Third, data about the time needed for IABP insertion or door‐to‐balloon time were not available in most of the studies. Finally, most of the eligible studies reported in‐hospital or 30‐day mortality, and long‐term data with more than 6 months were limited.

## 5 CONCLUSIONS

In patients with AMI complicated by CS undergoing primary PCI, the timing of initiation of IABP therapy does not appear to impact short‐term and long‐term clinical outcomes. However, this result should be interpreted with caution based on observational data. Appropriately, powered randomized trials are warranted to investigate the relative benefit of the two strategies, that is, IABP inserted before or after primary PCI in the future.

## CONFLICT OF INTEREST

The authors declare no potential conflict of interests.

## Supporting information


**Figure S1** Study selection flow diagram.
**Figure S2** Separate analysis of studies that enrolled patients with ST‐segment elevation myocardial infarction.
**Figure S3** Separate analysis of studies that published in full text.
**Figure S4** Funnel plot for (A) detection of publication bias and (B) Trim‐and‐Fill correction for publication bias for major bleeding.
**Table S1** The adjusted variables in each study.
**Table S2** Quality assessment of observational studies.
**Table S3** Quality assessment of randomized controlled trials.
