# Supplementary material for: Timing of initiation of intra‐aortic balloon pump in patients with acute myocardial infarction complicated by cardiogenic shock: A meta‐analysis
Source: Clin Cardiol. 2019 Sep 11;42(11):1126–34. doi: 10.1002/clc.23264 (PMC6837021; doi:10.1002/clc.23264)
Supplement: Supplementary file 1 — Figure S1 Study selection flow diagram. Figure S2 Separate analysis of studies that enrolled patients with ST‐segment elevation myocardial infarction. Figure S3 Separate analysis of studies that published in full text. Figure S4 Funnel plot for (A) detection of publication bias and (B) Trim‐and‐Fill correction for publication bias for major bleeding. Table S1 The adjusted variables in each study. Table S2 Quality assessment of observational studies. Table S3 Quality assessment of randomized controlled trials. [file CLC-42-1126-s001.docx]

**Supplementary Materials**

**Figure S1** Study selection flow diagram.

**Figure S2** Separate analysis of studies that enrolled patients with ST-segment elevation myocardial infarction.

**Figure S3** Separate analysis of studies that published in full text.

**Figure S4** Funnel plot for (A) detection of publication bias and (B) Trim-and-Fill correction for publication bias for major bleeding.

**Table S1** The adjusted variables in each study.

**Table S2** Quality assessment of observational studies.

**Table S3** Quality assessment of randomized controlled trials.

**Figure S1 Study selection flow diagram.**

**

**

ACS, acute coronary syndrome; IABP, intra-aortic balloon pump.

**Figure S2 Separate analysis of studies that enrolled patients with ST-segment elevation myocardial infarction.
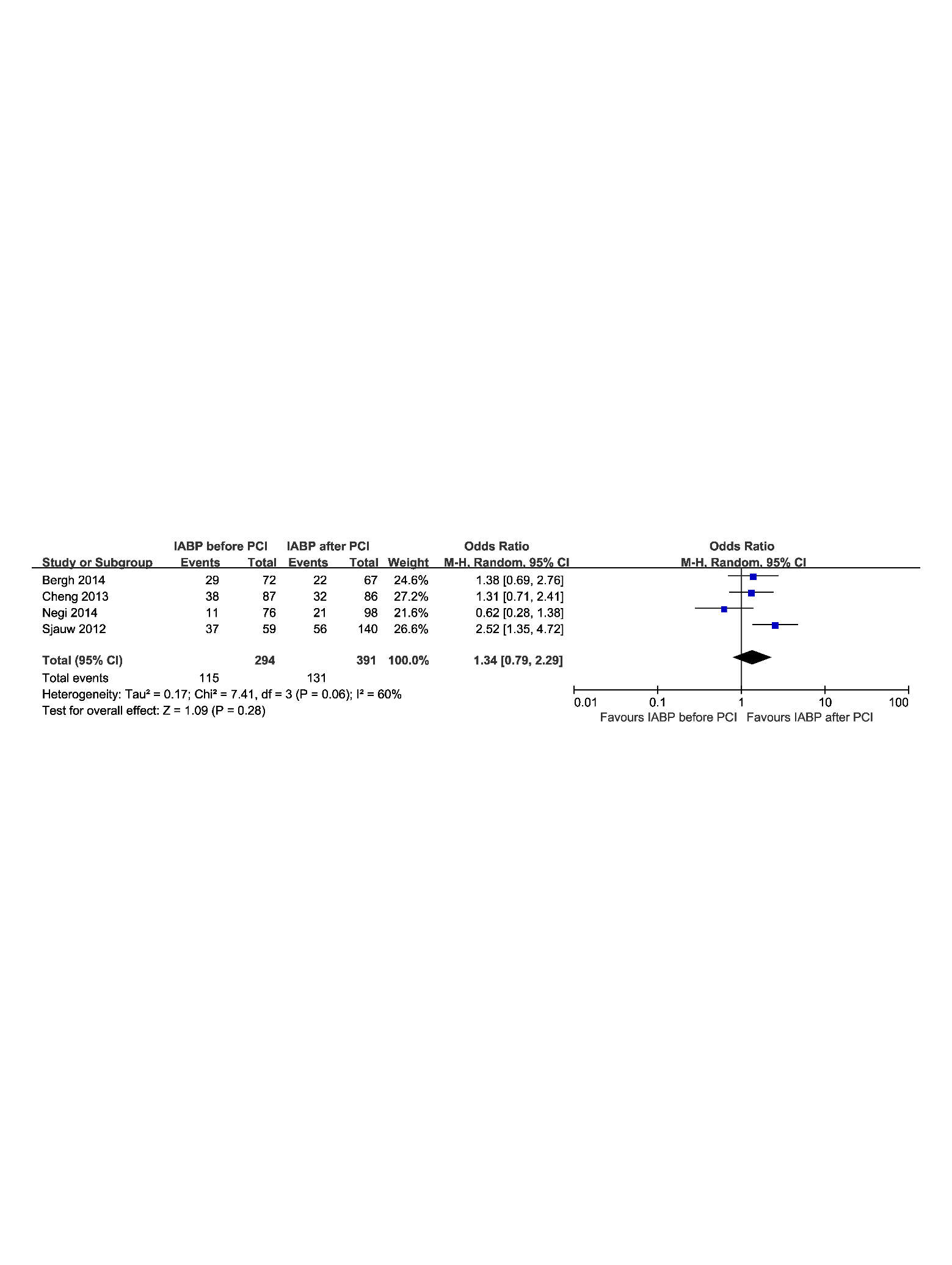
**

IABP, intra-aortic balloon pump; CI, confidence interval; PCI, percutaneous coronary intervention.

**Figure S3 Separate analysis of studies that published in full text.
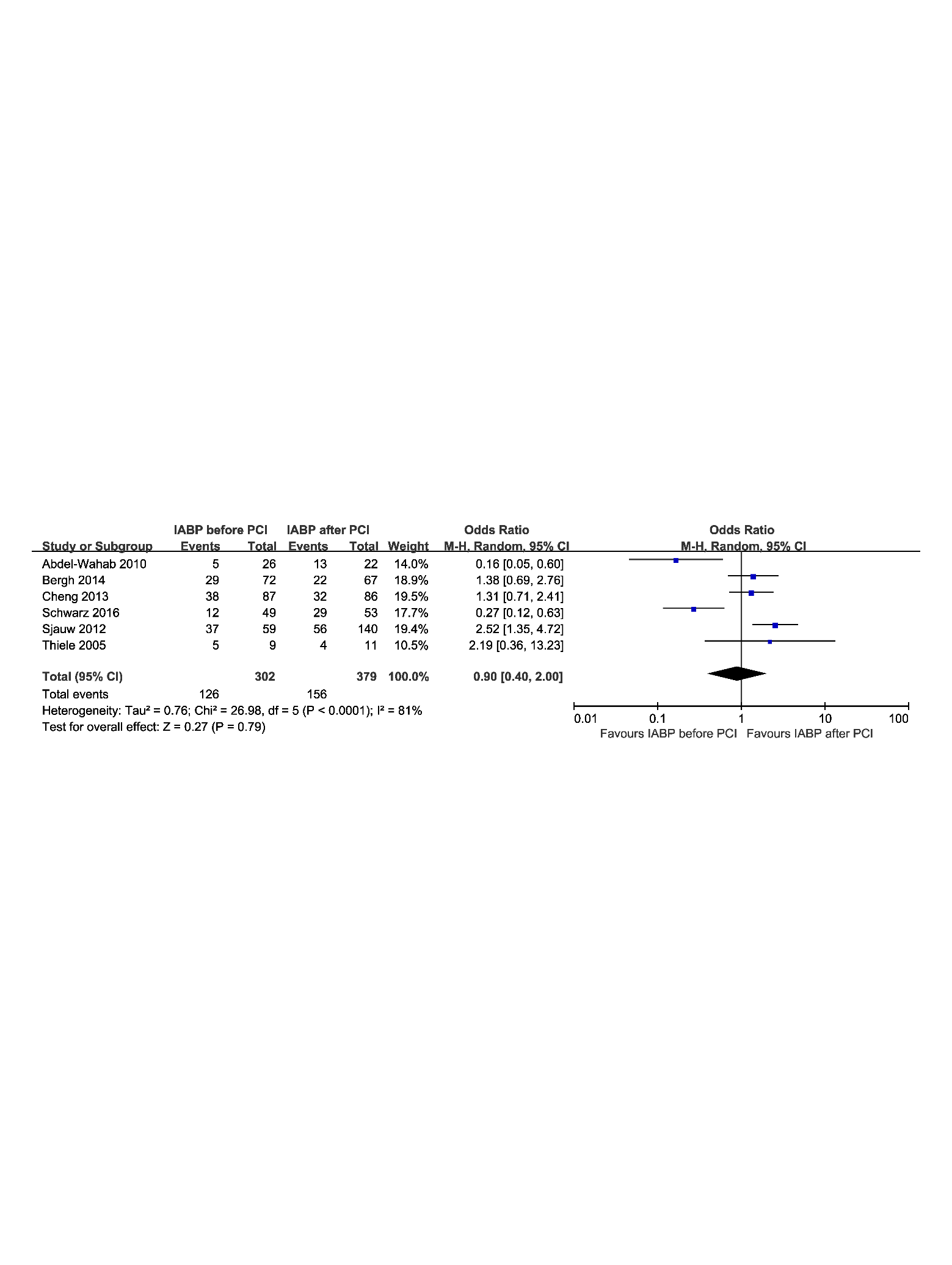
**

IABP, intra-aortic balloon pump; CI, confidence interval; PCI, percutaneous coronary intervention.

**Figure S4 Funnel plot for (A) detection of publication bias and (B) Trim-and-Fill correction for publication bias for major bleeding.**

**A**

**B**

**Table S1 The adjusted variables in each study.**

| Study | No. patients |
| --- | --- |
| Thiele 2005 | NA |
| Abdel-Wahab 2010 | Renal failure, age, systolic blood pressure, heart rate, ST-segment myocardial infarction,  glycoprotein IIB/IIIa inhibitors, baseline creatine kinase, mechanical ventilation, post PCI  Thrombolysis In Myocardial Infarction flow, multivessel disease, peak creatine kinase; |
| Sjauw 2012 | Age, admission creatinine, admission glucose, mean blood pressure, heart rate, left anterior  descending coronary artery related infarction, multivessel disease; |
| Cheng 2013 | Age, sex, history of myocardial infarction, out-of-hospital cardiac arrest, mechanical  ventilation, systolic blood pressure, location of myocardial infarction, culprit lesion in left  main coronary artery; |
| Bergh 2014 | Age, sex, body mass index, diabetes, hypertension, smoking, glycoprotein IIB/IIIa inhibitors,  bivalirudin, clopidogrel, unfractionated heparin, low-molecular weight heparins,  revascularization success, completeness of revascularization; |
| Negi 2014 | NA; |
| Schwarz 2016 | Acute renal failure, vasopressor use, resuscitation before PCI, age; |
| Yuan 2016 | NA; |
| Fuernau 2017 | Age, sex, need for invasive ventilation, prior stroke, peripheral artery disease, hypertension,  diabetes, prior medication, kidney function, hemoglobin values, Electrocardiograph findings,  extent of coronary artery disease, serum lactate and Thrombolysis In Myocardial Infarction  flow grades prior and post primary PCI timing of intra-aortic balloon pump implantation; |

NA, not appliable; PCI, percutaneous coronary intervention.

**Table S2 Quality assessment of observational studies.**

| Study | Selection | Comparability | Outcome | Total score |
| --- | --- | --- | --- | --- |
| Abdel-Wahab 2010 | 4 | 2 | 2 | 8 |
| Sjauw 2012 | 4 | 2 | 2 | 8 |
| Cheng 2013 | 4 | 2 | 3 | 9 |
| Bergh 2014 | 4 | 2 | 2 | 8 |
| Negi 2014 | NA | NA | NA | NA |
| Schwarz 2016 | 4 | 2 | 2 | 8 |
| Yuan 2016 | 4 | 2 | 3 | 9 |

NA, not appliable.

**Table S3 Quality assessment of randomized controlled trials.**

| Study | Random sequence generation  (selection bias) | Allocation concealment (selection bias) | Blinding of participants and personnel (performance bias) | Blinding of outcome assessment  (detection bias) | Incomplete outcome data  (attrition bias) | Selective reporting  (reporting bias) | Other bias |
| --- | --- | --- | --- | --- | --- | --- | --- |
| Thiele 2005 | Low risk | Low risk | High risk | High risk | Low risk | Low risk | Low risk |
| Fuernau 2017 | Low risk | Low risk | High risk | Low risk | Low risk | Low risk | Low risk |
